# Early Flowering (*ELF*) Gene Integrates Vegetative Growth, Flowering Regulation, and Reproductive Development in *Arabidopsis thaliana*

**DOI:** 10.3390/ijms27125615

**Published:** 2026-06-22

**Authors:** Rahmatullah Jan, Shahzad Iqbal, Sajad Ali, Mohammed A. Almalki, Mohammad Alfredan, Rashid Ismael Hag Ibrahim, Sajjad Asaf, Kyung-Min Kim

**Affiliations:** 1 Coastal Agriculture Research Institute, Kyungpook National University, Daegu 41566, Republic of Korea; rahmat2021@knu.ac.kr; 2 Department of Semiconductor Engineering, Gachon University, Seongnamdaero, Sujeong-gu, Seongnam-si 13120, Republic of Korea; 3 Department of Biological Sciences, College of Science, King Faisal University, Al-Ahsa 31982, Saudi Arabiamalmalki@kfu.edu.sa (M.A.A.);; 4 Department of Applied Biosciences, Graduate School, Kyungpook National University, Daegu 41566, Republic of Korea

**Keywords:** *Arabidopsis thaliana*, CRISPR/Cas9, early flowering gene, flowering regulation, reproductive development

## Abstract

Early flowering-related factors play pivotal roles in coordinating plant growth and reproductive development. In this study, we investigated the biological function of early flowering gene (*ELF*) in *Arabidopsis thaliana* using CRISPR/Cas9-mediated genome editing and construction of overexpression approaches. Two independent *ELF* overexpression (*OE-ELF*) and genome-edited (*ge-elf*) lines were generated and systemically analyzed. *ELF* overexpression significantly enhanced early seedling performance, increasing germination rate and seedling fresh weight by up to 8.7%, while genome-edited lines exhibited a marked reduction. Root growth was strongly promoted in *OE-ELF* plants, with root length increase of 85% and 75%, whereas *ge-elf* lines showed a reduction of up to 48%. At later developmental stages, *OE-ELF* plants displayed enhanced vegetative growth, including increased leaf length (32%), leaf area (91%), and accelerated flowering (21% earlier than wild type). In contrast, *ge-elf* delayed flowering by up to 25% and resulted in compact plant architecture. Reproductive development was severely compromised in *ge-elf* plants, which exhibited malformed inflorescences, reduced pollen germination, shortened silique (45%), and a drastic decrease in seed number per silique (70%). Conversely, *OE-ELF* plants showed increased silique number and seed per silique. Molecular analysis revealed that *ELF* positively regulates key flowering-related genes, including *FLC*, *SOC1*, *AP1*, and *LFY*, which correlated strongly with growth and reproductive traits. Our results demonstrate that *ELF* functions as a central regulator integrating vegetative growth, floral development, male fertility, and seed production in *Arabidopsis thaliana*.

## 1. Introduction

The transition from vegetative growth to flowering represents a critical developmental switch in the plant life cycle and is essential for successful reproduction and species survival. Precise regulation of flowering time ensures that reproductive development occurs under favorable environmental conditions, thereby maximizing fertility and seed production [1]. This transition is tightly coordinated with vegetative growth, floral organ development, and reproductive competence, highlighting the importance of integrated regulatory networks that balance between growth and reproduction [2,3].

The *Arabidopsis* has served as a powerful model system for dissecting the molecular mechanisms underlying flowering regulation due to its short life cycle, well-annotated genome, and extensive genetic resources. Genetic and molecular studies have demonstrated that the transition of flowering is influenced by multiple interconnected pathways that integrate environmental cues and endogenous developmental signals, including photoperiod, vernalization, autonomous, and gibberellin-dependent pathways [1,4]. These pathways converge on a set of key floral integrator genes, such as Flowering Locus C (*FLC*), Suppressor of Overexpression of CONSTANS 1 (*SOC1*), LEAFY (*LFY*), and APETALA 1 (*AP1*), which orchestrate floral transition and floral meristem identity [1]. Proper regulation of these genes is essential not only for flowering but also for normal floral organ development and reproductive success [5].

Early flowering-related factors have emerged as critical components of the flowering regulatory network, functioning at the interface of environmental signal perception, circadian clock regulation, and developmental phase transitions [6,7]. Genetic analysis of early flowering mutants in *Arabidopsis* has revealed that several *ELF*-associated regulators act as pleiotropic developmental factors, influencing not only the timing of floral transition but also vegetative growth patterns and reproductive development through modulation of key flowering repressors such as FLOWERING LOCUS (*FLC*) [6,8]. These findings suggest that early flowering gene regulators often operated upstream of, or in parallel with, major floral integrator genes, thereby exerting broad control over developmental programs. *ELF-related* proteins have been shown to participate in regulatory modules linking the circadian clock with photoperiod and hormone signaling pathways. For instance, components of the early flowering pathway have been implicated in circadian clock function and light signaling, acting downstream of Phytochrome-mediated inputs and influencing the temporal expression of flowering-time genes [9]. In addition, *ELF*-associated regulators have been reported to connect photoperiodic control with gibberellin signaling, thereby coordinating environmental responsiveness with endogenous growth cues [4]. Despite these advances, the biological roles of many *ELF*-related factors remain incompletely defined. In particular, limited information is available regarding how individual *ELF* genes influence vegetative growth dynamics, floral organ morphogenesis, male fertility, and seed development, or whether their functions extend beyond flowering time regulation to broader coordination of growth and reproductive competence. Given that related early flowering regulators (*LFY*) often exhibit pleiotropic phenotypes affecting meristem identity, circadian rhythms, and reproductive success [4], it remains unclear whether newly identified *ELF* genes function primarily as timing regulators or act as integrators linking circadian, hormonal, and developmental pathways. Addressing this knowledge gap is essential for understanding how flowering-time networks are coupled to plant growth and reproductive performance.

Recent advances in functional genomics have provided powerful tools to dissect gene function in plants. CRISPR/Cas9-mediated genome editing enables precise and heritable loss-of-function mutations, while constitutive overexpression approaches allow assessment of gene activity under elevated expression levels [10,11]. The combined use of these complementary strategies offers a robust framework for defining gene function and establishing causal relationships between gene expression and phenotypic outcomes. In this study, we employed both CRISPR/Cas9-mediated genome editing and constitutive overexpression approaches to systematically investigate the biological role of *ELF* in *Arabidopsis thaliana*. Two independent *ELF* overexpression lines and two genome-edited lines were generated and subjected to phenotypic, reproductive, and molecular analyses. We examined the effect of altered *ELF* expression on early seedling growth, vegetative development, flowering time, floral organ morphology, pollen germination, silique formation, and seed production. In addition, we analyzed the expression of key flowering-related genes, including *FLC*, *SOC1*, *AP1*, and *LFY*, to elucidate the molecular pathway associated with *ELF* function. Our findings reveal that *ELF* acts as a central regulator coordinating vegetative growth, floral development, male fertility, and seed formation in *Arabidopsis thaliana*. This study provides new insights into the multifunctional role of *ELF* and expands our understanding of the molecular mechanisms linking growth regulation with reproductive success in plants.

## 2. Results

### 2.1. Phenotypic Characterization of ELF Overexpression and Genome-Edited Lines

**Figure 1:**
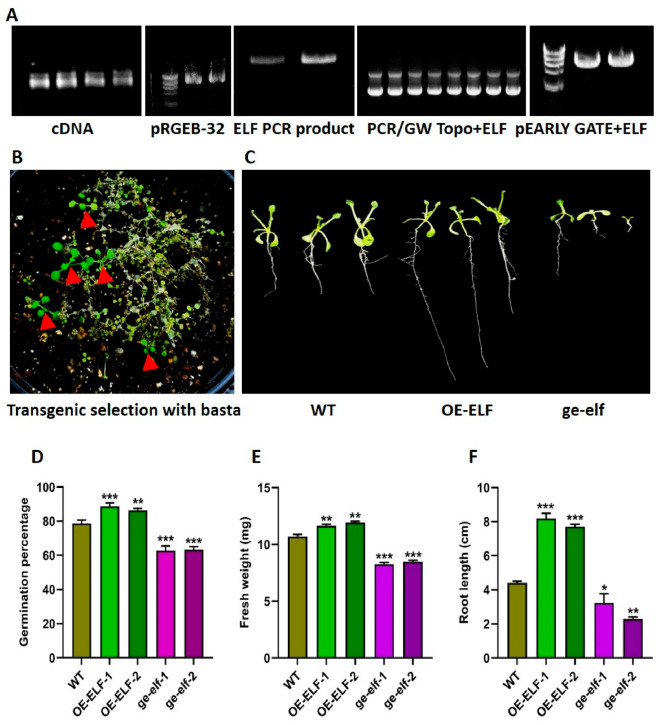
Generation and early phenotypic characterization of *ELF* overexpression and genome-edited *Arabidopsis* lines. (**A**) Molecular confirmation of *ELF* cloning and construct generation, showing cDNA amplification, pRGEB-32 vector, *ELF* PCR product, Gateway cloning of *ELF* into the entry vector, and final *pEARLY GATE-ELF* expression construct. (**B**) Selection of transgenic *Arabidopsis* seedlings via Basta spray, with red arrowheads indicating resistant seedlings. (**C**) Representative seedling phenotypes of WT, *ELF* overexpression (*OE-ELF-1*, *OE-ELF-2*), and genome-edited (*ge-elf-1*, *ge-elf-2*) lines. Quantitative analyses of germination percentage (**D**), fresh weight (**E**), and primary root length (**F**) of WT, *OE-ELF*, and *ge-elf* seedlings. Data represent mean ± SD from at least three independent biological replicates. Statistical significance was determined by one-way ANOVA followed by Bonferroni’s multiple comparison test; asterisks indicate significant differences compared with Col-0 (* represent *p* < 0.05, ** represent *p* < 0.01, *** represent *p* < 0.001).

To functionally evaluate *ELF*, two sgRNAs were designed and cloned into the pRGEB-32 vector (Figure S1A). The CRISPR-Cas9 construct, containing Cas9 driven by the Ubi promoter, the sgRNA expression cassette, and a hygromycin resistance marker, was assembled as illustrated in Figure S1A. The overall workflow for generating transgenic lines is shown in Figure S1B. Successful cDNA synthesis, sgRNA insertion into pRGEB-32, amplification of the full-length *ELF* coding sequence, generation of GW-TOPO recombinants, and construction of the final pEARLY-GATE overexpression vector were all verified by PCR followed by gel electrophoresis (Figure 1A).

Transformed T1 seedlings of the overexpression lines were screened by Basta application, and resistant individuals were selected for further analysis (Figure 1B). Phenotypic evaluation of 7-day-old seedlings revealed clear differences among the genotypes (Figure 1C). The overexpression lines (*OE-ELF-1* and *OE-ELF-2*) exhibited a significant increase in germination percentage, whereas the genome-edited lines (*ge-elf-1* and *ge-elf-2*) showed a significant reduction compared with wild-type plants (Figure 1D).

Consistently, analysis of seedling fresh weight one week after germination demonstrated a 8.7% increase in *OE-ELF-1*, while fresh weight was reduced by 22.8% and 20.8% in *ge-elf-1* and *ge-elf-2*, respectively, relative to the wild type (Figure 1E). Furthermore, root growth analysis showed that *ELF* overexpression significantly enhanced root length by 85% and 75% in *OE-ELF-1* and *OE-ELF-2*, respectively. In contrast, root growth was markedly suppressed in the genome-edited lines, with reductions of 26% in *ge-elf-1* and 48% in *ge-elf-2* compared with wild-type plants (Figure 1F). These results demonstrate that *ELF* positively regulates seed germination, seedling growth, and root development. Enhanced growth in *ELF* overexpression lines and growth suppression in genome-edited lines confirm that *ELF* is essential for early plant development.

### 2.2. ELF Regulates Vegetative Growth and Flowering Time in Arabidopsis

**Figure 2:**
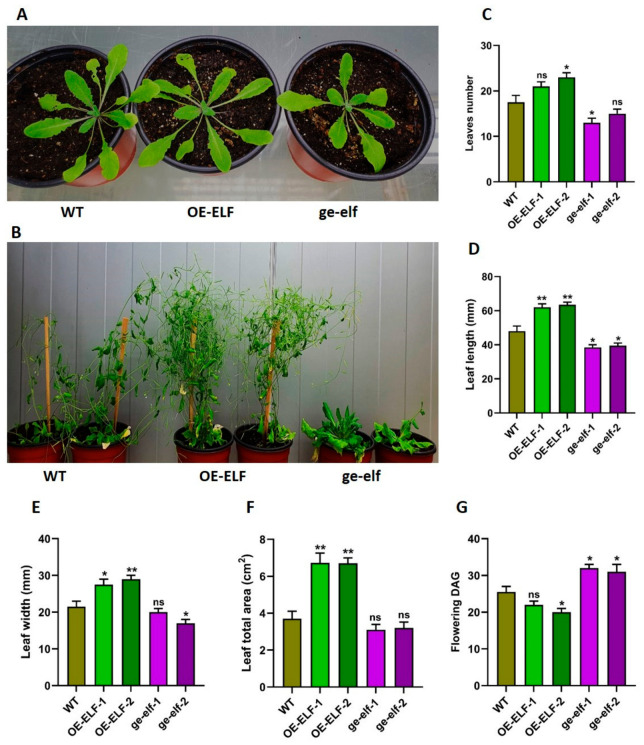
Phenotypic characterization of *ELF* overexpression and genome-edited *Arabidopsis* lines. Representative rosette phenotypes of wild-type Col-0, overexpression (*OE-ELF*), and genome-edited (*ge-elf*) plants at the vegetative stage after 20 days of germination are shown in (**A**). Adult plant phenotypes 40 days after germination, illustrating overall growth architecture of WT-1, WT-2, *OE-ELF-1*, *OE-ELF-2*, *ge-elf-1*, and *ge-elf-2* lines, are presented in (**B**). Quantitative analyses include leaf number per plant (**C**), leaf length (**D**), leaf width (**E**), total leaf area (**F**), and flowering time measured as days after germination (DAG) (**G**). Data represent mean ± SD from three biological replicates. Asterisks indicate statistically significant differences among genotypes as determined by one-way ANOVA followed by Bonferroni’s multiple comparison test (* represent *p* < 0.05, ** represent *p* < 0.01), while “ns” denotes no significant difference.

To elucidate the functional role of *ELF*, we compared the phenotypes of *ELF* overexpression and genome-edited lines with wild-type Col-0 plants. No obvious differences in vegetative growth were observed among the genotypes at 20 days after germination (Figure 2A). However, one month after germination, *OE-ELF-2* plants exhibited enhanced rosette growth and a significant increase in leaf number by 31%, whereas *ge-elf-1* plants showed a significant reduction in leaf number by 25% relative to wild-type plants (Figure 2C).

At the adult stage (40 days after germination), *ELF* overexpression resulted in pronounced leaf elongation and increased overall plant stature, whereas genome-edited plants displayed a compact phenotype with shorter leaves and reduced shoot length (Figure 2B). Quantitative analysis revealed that leaf length increased by 29% and 32% in *OE-ELF-1* and *OE-ELF-2* plants, respectively, while it decreased by 19% and 17% in *ge-elf-1* and *ge-elf-2* plants, respectively, compared with wild-type plants (Figure 2D). Similarly, leaf width was significantly increased by 27% and 34% in both the overexpression lines, whereas *ge-elf-2* plants exhibited a significant reduction of 20% relative to the wild type (Figure 2E).

As a consequence of these changes in leaf dimensions, total leaf area was markedly increased by 64% in *OE-ELF-1* and by 91% in *OE-ELF-2* plants, whereas no significant reduction in total leaf area was observed in *ge-elf-1* or *ge-elf-2* plants (Figure 2F). Analysis of flowering time demonstrated that *ELF* overexpression significantly accelerated flowering, with *OE-ELF-2* plants flowering 21% earlier than wild-type plants. In contrast, *ge-elf-1* and *ge-elf-2* plants flowered significantly later, exhibiting delays of 25% and 21%, respectively, compared with the wild type (Figure 2G). These results demonstrate that *ELF* positively regulates vegetative growth and promotes the transition to flowering, whereas loss of *ELF* function suppresses plant growth and delays reproductive development.

### 2.3. ELF Regulates Floral Organ Development and Male Reproductive Competence

**Figure 3:**
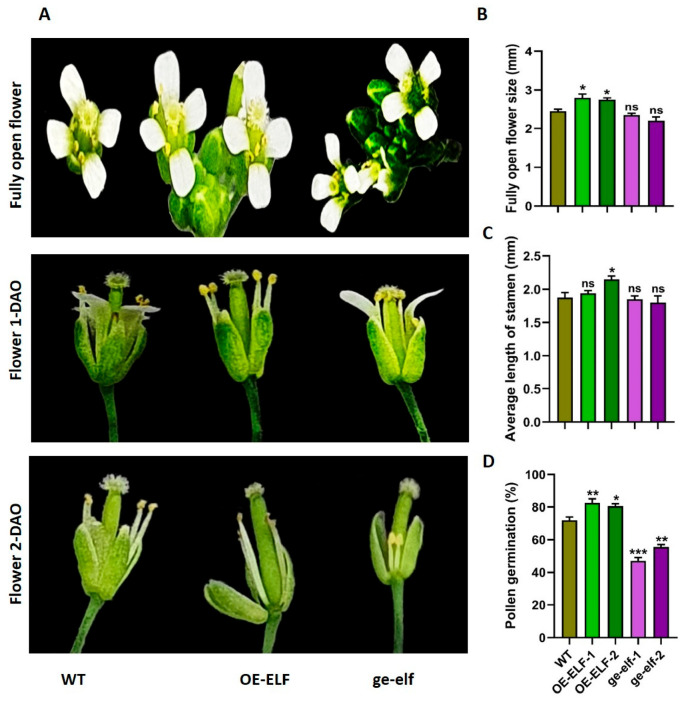
*ELF* influences floral morphology, organ growth, and pollen germination. (**A**) Representative images of fully open flowers, flowers at 1 day after opening (DAO), and flowers at 2 days after opening (DAO) from WT, *OE-ELF*, and *ge-elf* plants. (**B**) Fully open flower size. (**C**) Average stamen length measured in fully open flowers. (**D**) Pollen germination percentage in the indicated genotypes. Data represent mean ± SD from three independent biological replicates. Statistical significance was determined using one-way ANOVA followed by Bonferroni’s multiple comparison test; asterisks indicate significant differences compared with WT (* represent *p* < 0.05, ** represent *p* < 0.01, *** represent *p* < 0.001), while “ns” denotes no significant difference.

Phenotypic analysis of floral development revealed clear morphological and functional differences among WT, *OE-ELF*, and *ge-elf* lines (Figure 3). Flowers were imaged and analyzed six hours after flower opening. At this stage, flowers from *OE-ELF* plants appeared larger and more robust than those of WT plants, whereas *ge-elf* flowers were visibly smaller and developmentally compromised (Figure 3A). These phenotypic differences were already apparent at early post-anthesis stages (1 and 2 days after flower opening), indicating that *ELF* influences floral organ growth and maturation throughout development. Quantitative measurements supported these observations. Flowers from *OE-ELF-1* and *OE-ELF-2* plants were significantly larger than those of WT plants, exhibiting increases of 14% and 12%, respectively (Figure 3B). In contrast, genome-edited lines displayed a trend toward reduced flower size; however, this reduction was not statistically significant compared with WT plants, suggesting a positive contribution of *ELF* to overall floral growth. Stamen length showed a modest but significant increase of 14% in the *OE-ELF-2* line relative to WT plants (Figure 3C), whereas genome-edited lines exhibited no significant differences.

Notably, pollen germination efficiency was strongly dependent on *ELF* expression levels (Figure 3D). *ELF* overexpression significantly enhanced pollen germination, with increases of 14% and 11% in *OE-ELF-1* and *OE-ELF-2* plants, respectively, compared with WT. In contrast, genome-edited lines showed a pronounced reduction in pollen germination, decreasing to 34% and 22% in *ge-elf-1* and *ge-elf-2* plants, respectively, relative to WT. These results indicate that *ELF* plays a critical role in male gametophyte viability. Overall, these findings demonstrate that *ELF* not only regulates the expression of flowering-related genes but also exerts a direct influence on floral organ growth and reproductive success, particularly by promoting flower size and pollen germination capacity.

### 2.4. ELF Influences Reproductive Development and Seed Yield in Arabidopsis

**Figure 4:**
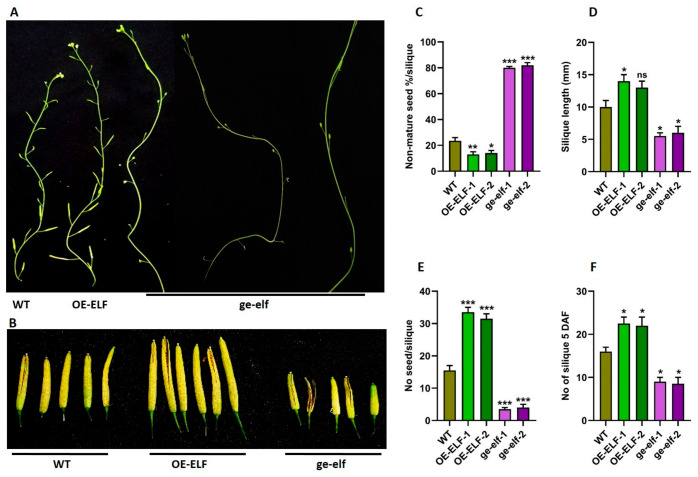
*ELF* regulates inflorescence development, silique morphology, and seed production in *Arabidopsis*. Representative inflorescence phenotypes of WT, *OE-ELF*, and *ge-elf* plants are shown in (**A**). Representative siliques from WT, *OE-ELF*, and *ge-elf* lines are presented in (**B**). Quantitative analyses include the percentage of non-mature seeds per silique (**C**), silique length (**D**), seed number per silique (**E**), and total number of siliques per plant (**F**). Data represent mean ± SD from three independent biological replicates. Statistical significance was determined using one-way ANOVA followed by Bonferroni’s multiple comparison test; asterisks indicate significant differences compared with WT (* represent *p* < 0.05, ** represent *p* < 0.01, *** represent *p* < 0.001), while “ns” denotes no significant difference.

To further assess the role of *ELF* in reproductive development, we examined inflorescence architecture, silique morphology, and seed production in *ELF* overexpression (*OE-ELF*) and genome-edited (*ge-elf*) lines in comparison with wild-type (WT) plants. Pronounced differences in reproductive traits were observed among the genotypes. *OE-ELF* plants exhibited elongated, healthy, and normally developed inflorescences, whereas genome-edited plants showed reduced inflorescence growth and overall weaker reproductive structures compared with WT plants (Figure 4A). Consistent with these observations, silique morphology and seed development were markedly affected (Figure 4B).

Quantitative analysis revealed that the percentage of non-mature seeds per silique decreased by 44% and 40% in *OE-ELF-1* and *OE-ELF-2* plants, respectively, whereas *ge-elf-1* and *ge-elf-2* lines exhibited dramatic increases of 240% and 248%, respectively, relative to WT plants (Figure 4C). Moreover, silique length increased significantly by 40% in *OE-ELF-1* plants, while *ge-elf-1* and *ge-elf-2* plants produced siliques that were approximately 45% and 40% shorter, respectively, compared with WT plants (Figure 4D). Correspondingly, seed number per silique increased by 116% and 103% in *OE-ELF-1* and *OE-ELF-2* plants, whereas it was reduced by 77% in *ge-elf-1* and by 74% in *ge-elf-2* plants relative to WT plants (Figure 4E).

In addition, the total number of siliques per plant increased by 40% in *OE-ELF-1* and by 73% in *OE-ELF-2* plants, while both the genome-edited lines produced 43% and 46% fewer siliques, respectively, compared with WT plants (Figure 4F). Collectively, these results demonstrate that *ELF* positively regulates reproductive development, silique formation, and seed yield, whereas loss of *ELF* function severely impairs fertility and reproductive success in *Arabidopsis*.

### 2.5. Loss of ELF Function Causes Severe Defects in Flower Development and Reproductive Success

**Figure 5:**
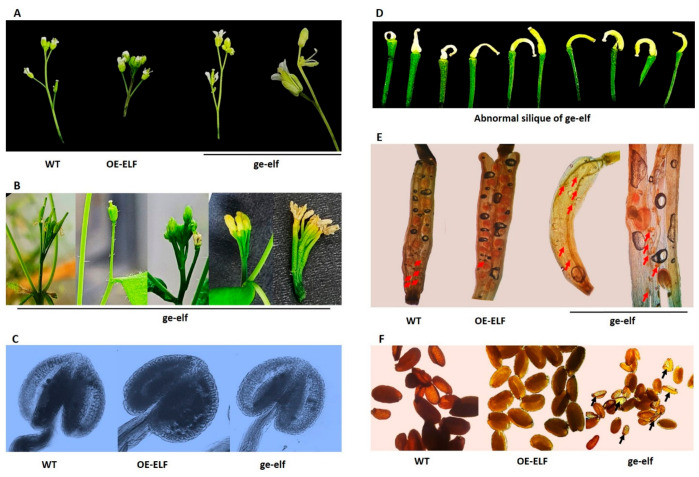
*ELF* regulates floral development, male fertility, silique formation, and seed development in *Arabidopsis*. (**A**) Representative inflorescences and flowers of WT, *OE-ELF*, and *ge-elf* plants. (**B**) Close-up views of developing flowers in *ge-elf* lines, showing abnormal organ elongation, partial tissue degeneration, and altered inflorescence architecture. (**C**) Microscopic analysis of stamens and pollen grains 12 h after flower opening. WT and *OE-ELF* plants show normal, well-developed pollen grains, whereas *ge-elf* plants exhibit reduced pollen development. (**D**) Representative siliques showing curved, shortened, and malformed siliques in *ge-elf* plants. (**E**) Tetrazolium assay highlighting abnormal ovule and seed development in *ge-elf* plants (red arrows), compared with normal seed arrangement in WT and *OE-ELF*. (**F**) Seeds morphology from Col-0, *OE-ELF*, and *ge-elf* plants, showing reduced well-developed seed number and abnormal seed morphology in *ge-elf* lines.

To further investigate the role of *ELF* in *Arabidopsis* reproductive development, we compared floral morphology, silique formation, and seed development among WT, *OE-ELF*, and *ge-elf* lines (Figure 5). Visual inspection of inflorescences revealed clear morphological differences among the genotypes (Figure 5A). WT plants exhibited normal floral architecture, whereas *OE-ELF* plants displayed more robust and healthy floral structures. In contrast, *ge-elf* plants showed pronounced abnormalities, including reduced flower size and distorted floral organs, indicating compromised reproductive development. Closer examination of developing flowers further confirmed these phenotypic differences (Figure 5B). Flowers from *ge-elf* plants exhibited abnormal organ elongation and partial degeneration of reproductive tissues. In some *ge-elf* plants, a single flower developed in place of a normal inflorescence, while others displayed compact and jointed inflorescences, reflecting disrupted inflorescence architecture.

Microscopic analysis of stamen and pollen grain development demonstrated that *ELF* is essential for normal male reproductive development (Figure 5C). In WT and *OE-ELF* plants, stamens showed normal anther morphology with well-developed pollen grains. In contrast, *ge-elf* plants exhibited markedly reduced pollen development, as analyzed 12 h after flower opening.

Consistent with the observed floral defects and reduced pollen development, silique formation was severely impaired in *ge-elf* plants. Siliques in *ge-elf* lines were frequently curved, shortened, and malformed (Figure 5D). Histological analysis of siliques further revealed significant differences in seed fertilization and development among the genotypes (Figure 5E). WT and *OE-ELF* siliques predominantly contained fertilized and normally developing seeds, whereas *ge-elf* siliques showed a high proportion of non-fertilized, non-developed ovules and only a few developing seeds. As a consequence of these defects, overall seed development and seed set were markedly reduced in *ge-elf* plants and enhanced in *OE-ELF* plants relative to WT (Figure 5F). Collectively, these results demonstrate that *ELF* plays a critical role in floral development, silique integrity, male fertility, and successful seed formation, and that loss of *ELF* function leads to severe reproductive defects in *Arabidopsis*.

### 2.6. ELF Gene Expression Level in Different Tissues

**Figure 6:**
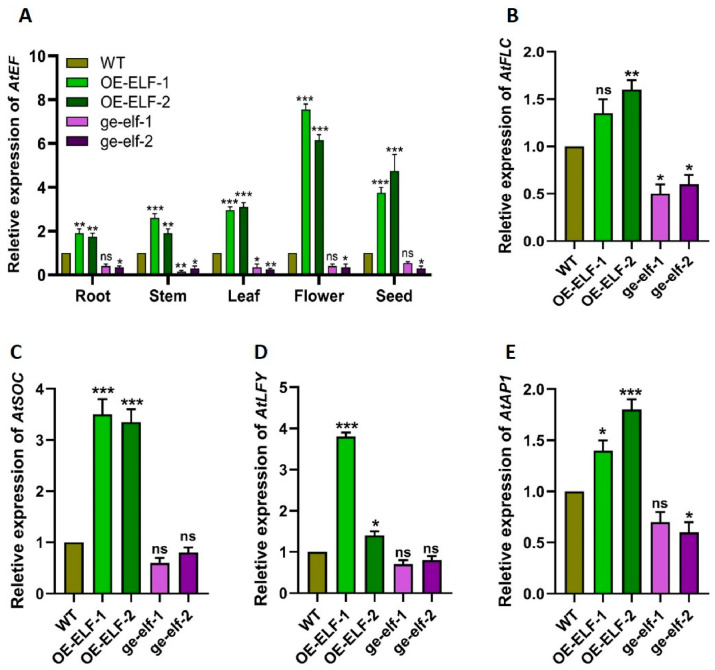
Expression profiling of *ELF* and flowering-related genes in *Arabidopsis* transgenic lines. (**A**) Relative expression of *ELF* in root, stem, leaf, flower, and seed tissues of WT, *OE-ELF-1*, *OE-ELF-2*, *ge-elf-1*, and *ge-elf-2*. (**B**–**E**) Relative expression levels of *AtFLC* (**B**), *AtSOC1* (**C**), *AtLFY* (**D**), and *AtAP1* (**E**) in the indicated genotypes. Data represent mean ± SD from three independent biological replicates. Statistical significance was determined using one-way ANOVA followed by Bonferroni’s multiple comparison test; asterisks indicate significant differences compared with WT (* represent *p* < 0.05, ** represent *p* < 0.01, *** represent *p* < 0.001)), while “ns” denotes no significant difference.

Quantitative expression analysis revealed that *ELF* is broadly expressed across different organs, and its transcript abundance is markedly altered in transgenic lines compared with WT plants (Figure 6A). In *ELF* overexpression lines, transcript levels were significantly elevated in all tissues, with the strongest induction observed in reproductive organs relative to WT. In contrast, genome-edited *ELF* lines exhibited a consistent reduction in *ELF* expression across all examined organs. Consistent with enhanced *ELF* activity, *AtFLC* expression was significantly increased in the *OE-ELF-2 line*, whereas both genome-edited lines showed a significant reduction (Figure 6B). Relative to WT, *AtFLC* transcript levels increased by approximately 60% in *OE-ELF-2*, but decreased by 50% in *ge-elf-1* and 40% in *ge-elf-2* plants, indicating a positive association between *ELF* and *FLC* transcript accumulation.

Downstream flowering regulators displayed differential responses to altered *ELF* expression. *AtSOC1* was strongly induced in both *OE-ELF-1* and *OE-ELF-2* lines (Figure 6C), showing increases of approximately 250% and 235%, respectively, compared with WT, while no significant change was detected in the genome-edited lines. Similarly, *AtLFY* expression increased in both overexpression lines but was reduced in the genome-edited lines (Figure 6D). In addition, *AtAP1* expression was significantly upregulated by *ELF* overexpression (Figure 6E), with increases of 39% and 80% in *OE-ELF-1* and *OE-ELF-2*, respectively, while a significant 40% reduction was observed in *ge-elf-2* plants relative to WT. Collectively, these results demonstrate that *ELF* acts as a positive regulator of multiple flowering-related genes, with the strongest effects observed in constitutive overexpression backgrounds.

### 2.7. Correlation Analysis of Growth, Reproductive, and Molecular Traits

**Figure 7:**
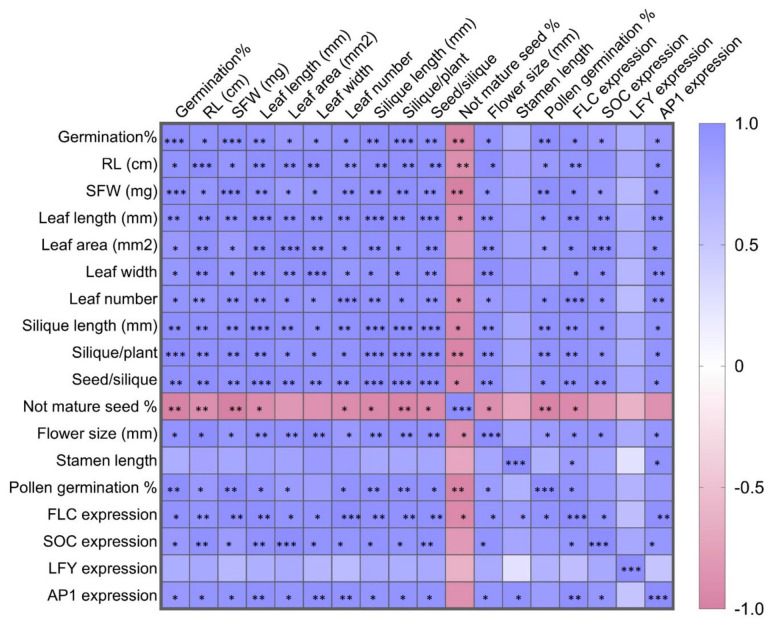
Correlation analysis of vegetative growth, reproductive traits, and flowering-related gene expression. Pearson’s correlation heatmap showing relationships among germination percentage, root length (RL), shoot fresh weight (SFW), leaf morphological traits, silique characteristics, seed development parameters, floral traits, pollen germination, and expression levels of flowering-related genes (*FLC*, *SOC*, *LFY*, and *AP1*). Blue colors indicate positive correlations, while red colors indicate negative correlations. The intensity of color reflects the strength of the correlation coefficient (r), with values ranging from −1 to +1. (* represent *p* < 0.05, ** represent *p* < 0.01, *** represent *p* < 0.001).

To examine the relationships among growth, reproductive, and molecular traits associated with *ELF* function, Pearson’s correlation analysis was performed using germination, vegetative growth parameters, reproductive traits, and flowering-related gene expression level (Figure 7). Strong positive correlation was observed among the vegetative growth traits, including root length, shoot fresh weight, leaf length, leaf area, leaf width, and leaf number, indicating coordinated regulation of plant growth. These vegetative parameters also showed strong positive correlations with reproductive traits such as silique length, number of silique per plant, seeds per silique, flower size, pollen germination percentage, and stamen length.

In contrast, the percentage of non-mature seeds exhibited strong negative correlations with most vegetative and reproductive traits, including germination rate, silique length, seeds per silique, pollen germination, and flower size, indicating that enhanced growth and reproductive performance are associated with reduced seed abortion. Notably, flowering-related gene expression levels, including *FLC*, *SOC*, and *AP1*, show strong positive correlations with vegetative growth traits, silique development, pollen germination, and seed set. *LFY* expression also displayed positive correlation with these traits, although the correlation strength was comparatively weaker than that of *FLC*, *SOC*, and *AP1*. Overall, the correlation analysis reveals a tightly coordinated relationship between *ELF*-associated growth regulation, reproductive success, and flowering gene expression, supporting the role of *ELF* as a central regulator linking vegetative vigor, male fertility, and seed development in *Arabidopsis*.

## 3. Discussion

**Figure 8:**
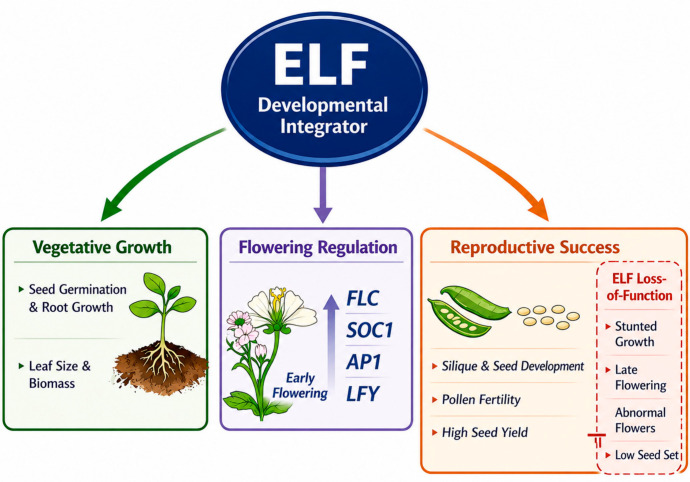
**Graphical abstract.** *ELF* acts as a central developmental integrator coordinating vegetative growth, flowering regulator, and reproductive development in *Arabidopsis*. Elevated *ELF* activity enhances seed germination, root growth, and leaf biomass, promotes flowering through upregulation of key floral regulators (*FLC*, *SOC1*, *AP1*, and *LFY*), and supports reproductive success by improving floral organ development, pollen fertility, silique formation, and seed yield. In contrast, loss of *ELF* function leads to reduced growth, delayed flowering, floral abnormalities, impaired male fertility, and low seed set.

Flowering time regulation is strongly interconnected with vegetative growth, floral organ development, and reproductive success, enabling plants to optimize fitness under fluctuating environmental conditions. In this study, we demonstrated that *ELF* functions as a central regulatory node coordinating early growth, flowering transition, floral development, male fertility, and seed production in *Arabidopsis*. By combining CRISPR/Cas9-mediated loss-of-function mutants with constitutive overexpression lines, our work reveals that *ELF* exerts pleiotropic effects extending well beyond flowering time control, integrating developmental and reproductive programs at both morphological and molecular levels. These integrative roles of *ELF* are summarized in the graphical abstract (Figure 8), which depicts *ELF* as a central developmental integrator linking vegetative growth, flowering time regulation, and reproductive success through coordinated phenotypic and molecular regulation.

Our results showed that *ELF* promoted seed germination, seedling biomass accumulation, and root elongation, as evidenced by enhanced growth in *OE-ELF* lines and severe growth inhibition in *ge-elf* lines. These findings indicate that *ELF* contributes to early developmental vigor, likely by modulating growth-related signaling pathways that operate prior to floral induction. Early seedling establishment is a critical determinant of later reproductive success, and genes that regulate flowering often play an additional role in vegetative development [12]. Although hormonal pathways were not directly examined in this study, the pronounced increase in root length observed in *OE-ELF* plants suggests, based on previous reports, that *ELF* may influence hormonal regulation, particularly gibberellin signaling, which is known to control both root elongation and flowering time [13]. Previous studies have shown that flowering regulators frequently interact with GA metabolism and signaling to coordinate vegetative growth with reproductive transition [14]. Thus, *ELF* may act upstream of, or in parallel with, hormone-mediated growth networks to ensure coordinated developmental progression.

Consistent with its role in early growth, *ELF* also positively regulates vegetative vigor and accelerates the floral transition. *OE-ELF* resulted in enhanced rosette expansion, increased leaf size, and earlier flowering, whereas *ge-elf* plants caused compact growth and delayed flowering. These phenotypes strongly support a positive role of *ELF* in vegetative vigor and floral induction. Leaf number and leaf area are linked to flowering time, as the shoot apical meristem integrates age-dependent signals with environmental cues to initiate reproduction [15]. The increased leaf area observed in *OE-ELF* plants likely enhances photosynthetic capacity, providing additional resources that support earlier and more robust reproductive development. At the molecular level, *ELF* overexpression was associated with elevated expression of major floral integrators, including *SOC1*, *AP1*, and *LFY*, which indicates that *ELF* acts upstream of these genes or modulates their transcriptional activity. *SOC1* functions as a central hub integrating photoperiod, GA, and autonomous pathways [16], while *AP1* is essential for floral meristem identity and floral organ initiation [17]. The coordinated induction of these genes in *OE-ELF* plants provides a mechanistic explanation for the accelerated flowering and enhanced floral development observed in this study.

The positive association between *ELF* and *FLC* expression is particularly intriguing, as *FLC* is classically characterized as a floral repressor [18]. However, emerging evidence suggests that *FLC* can exert context-dependent functions beyond flowering repression, including roles in vegetative growth, reproductive development, and resource allocation [15,19]. Genome-wide analysis has further demonstrated that *FLC* directly regulates numerous target genes involved in diverse developmental pathways and can function as either a transcriptional repressor or activator, depending on the target gene and developmental context [20]. Interestingly, *AtFLC* expression was elevated in the *OE-ELF-2* line despite the early-flowering phenotype. These findings suggest that the relationship between FLC expression and flowering behavior may be more complex than predicted by the classical flowering model. Therefore, the accelerated flowering observed in *OE-ELF* plants may result from *ELF*-mediated activation of flowering-promoting pathways that outweigh the repressive influence of increased *FLC* expression. The simultaneous upregulation of *FLC* alongside downstream floral integrators such as *SOC1* in *OE-ELF* plants indicates that *ELF* likely fine-tunes flowering-time regulation rather than functioning as a simple binary switch. Such modulation may enable plants to balance vegetative growth, flowering competence, and developmental progression under varying internal conditions.

Beyond flowering initiation, our data demonstrate that *ELF* plays a critical role in floral organ growth and male reproductive competence. *ELF* overexpression increased flower size, stamen length, and pollen germination efficiency, whereas *ELF* loss-of-function resulted in severe floral abnormalities, impaired pollen development, and reduced male fertility. These findings highlight that *ELF* is not merely a flowering-time regulator but a key determinant of reproductive organ function. Male fertility is particularly sensitive to disruption in developmental timing, hormonal balance, and circadian regulation [21,22,23]. The drastic reduction in pollen germination in *ge-elf* lines suggests that *ELF* is required for proper anther development and pollen maturation. Several flowering-related genes, including *LFY* and *SOC1*, have been reported to influence reproductive organ identity and fertility when misexpressed [16,24,25]. Given the positive correlation between *ELF* expression and these integrators, *ELF* likely contributes to a transcriptional network that ensures synchronized development of floral organs and gametophytes.

A striking outcome of this study is the profound effect of *ELF* on silique formation and seed production. *ELF* overexpression increased silique length, silique number, and seed set, while *ELF* loss-of-function caused extensive seed abortion, malformed silique, and directly reduced fertility. These results highlight the importance of *ELF* in the post-fertilization process, including ovule fertilization, embryo development, and resource allocation to developing seeds. Seed yield is a complex trait influenced by the integration of vegetative growth capacity, floral fertility, and hormonal signaling, as shown by a study linking reproductive meristem activity and hormone regulation with seed production outcomes in *Arabidopsis* and crop species [26]. The strong negative correlation between non-mature seed and vegetative or reproductive traits suggests that *ELF*-mediated enhancement of growth and floral function directly translates into improved reproductive success. Similar pleiotropic effects have been reported for flowering regulators that also influence meristem maintenance and reproductive development [27].

Correlation analysis revealed strong positive relationships among vegetative growth traits, reproductive performance, and flowering gene expression, supporting a model in which *ELF* acts as a central integrator linking growth vigor with reproductive success. By coordinating seedling establishment, vegetative expansion, flowering induction, floral organ development, and fertility, *ELF* ensures a smooth developmental continuum from early growth to seed production. Such integrative regulators are increasingly recognized as key determinants of plant fitness, enabling plants to synchronize internal developmental programs with environmental signals. Our findings place *ELF* among this class of multifunctional regulators and expand the conceptual framework of flowering-time genes as broad developmental coordinators rather than isolated timing factors. Overall, our results support a model in which *ELF* acts as a central developmental integrator linking vegetative vigor with flowering regulation and reproductive success. This integrative role provides a conceptual framework for understanding how flowering time regulation coordinates multiple developmental dynamics in plants.

## 4. Materials and Methods

### 4.1. Generation of Transgenic Arabidopsis Plants Overexpressing ELF

*Arabidopsis thaliana* (Col-0) overexpression lines for *ELF* were generated by first amplifying the full-length coding region of *ELF* (Locus: AT3G21320) and inserted into the *pCR8/GW/TOPO* entry vector, which carries spectinomycin resistance. The construct was introduced into *Escherichia coli* DH5α via heat-shock transformation, and positive colonies were verified through colony PCR followed by sequencing. The confirmed entry clone was subsequently transferred into the Gateway-compatible binary vector *pEarleyGate103*, harboring a CaMV 35S promoter and kanamycin resistance, via an LR recombination reaction to achieve constitutive transgene expression. All resulting plasmids were validated by commercial sequencing prior to plant transformation. The finalized overexpression construct was electroporated into *Agrobacterium tumefaciens* strain GV3101, which was then used to transform *Arabidopsis* plants using the floral dip method [28]. Transgenic seedlings were selected by spraying 1-week-old progeny with 5 µg/mL Basta, and herbicide-resistant T1 lines were identified for further characterization.

### 4.2. Generation of ELF-Edited Arabidopsis via CRISPR/Cas9

To obtain *Arabidopsis thaliana* lines carrying targeted mutations in *ELF*, two sgRNAs were designed using the CRISPR RGEN design platform. Each sgRNA was assembled into the CRISPR/Cas9 expression vector *pRGEB32*, which contains the Cas9 nuclease, by inserting the guide sequences downstream of the U3 promoter through BsaI-based Golden Gate cloning [29]. Sequence analysis was performed to verify the correct incorporation of each guide RNA. The validated CRISPR constructs were introduced into *Agrobacterium tumefaciens* strain EHA105 using heat-shock transformation and subsequently delivered into *Arabidopsis* plants through the standard floral dip procedure. Putative T1 transformants were confirmed through genotypic analysis to identify individuals carrying CRISPR-induced edits at the *ELF* locus. Genome-edited lines confirmed through molecular analysis were selected for downstream characterization.

### 4.3. Molecular Confirmation and Sequencing of CRISPR-Edited Arabidopsis Lines

Genomic DNA was isolated from putative genome-edited *Arabidopsis* plants using the DNeasy Plant Mini Kit (QIAGEN, Cat. 69104, Hilden, Germany) according to the manufacturer’s protocol. The presence of T-DNA was verified by PCR amplification of the hygromycin resistance marker using the Hygro-F and Hygro-R primers. PCR reactions were carried out with high-fidelity Pfu polymerase in combination with the Genetbio PCR Master Mix (Yuseong-gu, Daejeon, Republic of Korea), following the recommended guidelines. To assess mutations introduced by CRISPR/Cas9, the coding region of *ELF* was amplified using gene-specific primers, and the resulting products were subjected to Sanger sequencing. Sequences were compared against the NCBI reference sequence to identify insertions, deletions, or other sequence alterations at the target site (Figure S2).

### 4.4. RNA Extraction, cDNA Synthesis and Quantitative Real-Time PCR Analysis

Total RNA was extracted using the RNeasy Plant Mini Kit (Qiagen, Valencia, CA, USA) following the manual instructions. A cDNA library was synthesized from 2 μg of total RNA using the qPCR-Bio cDNA Synthesis Kit (PCRBIOSYSTEM, Seoul, Republic of Korea), according to the manual instructions. qPCR was performed using the cDNA template and the StepOne™ Plus RT-PCR system (Thermo Fisher Scientific, Seoul, Republic of Korea). The reaction was performed with 2x Real-Time PCR Master Mix (including SYBR Green I: BIOFACT, Daejeon, Republic of Korea). *Actin* was used as the reference gene, and data were analyzed via the 2^–∆∆Ct^ method.

### 4.5. Plants and Growth Conditions

*Arabidopsis thaliana* plants used in this study belonged to the Columbia-0 (Col-0) ecotype. Three plant groups were analyzed: wild-type (WT; control), *AtELF* overexpression lines (*OE-ELF*), and genome-edited lines (*ge-elf*). For both the overexpression and genome-edited groups, two independent lines were used (*OE-ELF-T1* and *OE-ELF-T2*; *ge-elf-t1* and *ge-elf-t2*, respectively). Plants were grown under controlled environmental conditions with a 16 h light/8 h dark photoperiod, a constant temperature of 25 °C, and 60% relative humidity, as previously described [30].

### 4.6. Evaluation of Seed Viability Using Tetrazolium Assay

To assess seed viability across all genotypes, a tetrazolium chloride (TTC) (Alfa Aesar, Shore Road, Heysham, England), assay was performed following the protocol described by [31]. Siliques from each genotype were collected one week after flower opening and subjected to the tetrazolium assay. Intact siliques were incubated in a 1% (*w*/*v*) aqueous tetrazolium chloride solution and maintained at 30 °C for 2 h in the dark. During incubation, tetrazolium chloride was metabolically reduced by NADH-dependent reductase activity in viable tissues to form a red, insoluble formazan product, indicating seed viability.

### 4.7. Statistical Analysis

The experiment was conducted using a completely randomized design with three biological replicates. Data were analyzed using one-way analysis of variance (ANOVA), followed by Bonferroni’s multiple comparison test. Results are presented as mean ± standard deviation. All graphs were generated using GraphPad Prism 10 (GraphPad Software, San Diego, CA, USA).

## 5. Conclusions, Limitations, and Future Perspectives

This study demonstrated that *ELF* is a central regulator of growth, flowering, and reproductive success in *Arabidopsis thaliana*. *ELF* overexpression enhanced seed germination, vegetative growth, flowering transition, pollen germination, and seed yield, where CRISPR/Cas9-mediated loss of *ELF* function caused delayed flowering, impaired floral organ development, reduced male fertility, malformed siliques, and severe reductions in seed set. The coordinated upregulation of flowering-related genes in overexpression lines highlights *ELF* as an integrator of vegetative and reproductive developmental programs.

A limitation of this study is that *ELF* function was evaluated under control growth conditions, and its role under environmental stress remains unclear. In addition, although strong associations between *ELF* and downstream flowering-related genes were observed, the direct molecular mechanisms underlying these interactions were not addressed due to limited resources.

Future work should focus on identifying direct *ELF* targets and regulatory mechanisms using genome-wide approaches, as well as exploring *ELF* function under diverse environmental conditions and in crop species.

## Data Availability

All data supporting the findings of this study are included within the article.

## Supplementary Materials

The following supporting information can be downloaded at: https://www.mdpi.com/article/10.3390/ijms27125615/s1.
