# Supplementary material for: Early Flowering (ELF) Gene Integrates Vegetative Growth, Flowering Regulation, and Reproductive Development in Arabidopsis thaliana
Source: Int J Mol Sci. 2026 Jun 22;27(12):5615. doi: 10.3390/ijms27125615 (PMC13300733; doi:10.3390/ijms27125615)
Supplement: Supplementary file 1 [file ijms-27-05615-s001.zip › ijms-4330349-supplementary.pdf]

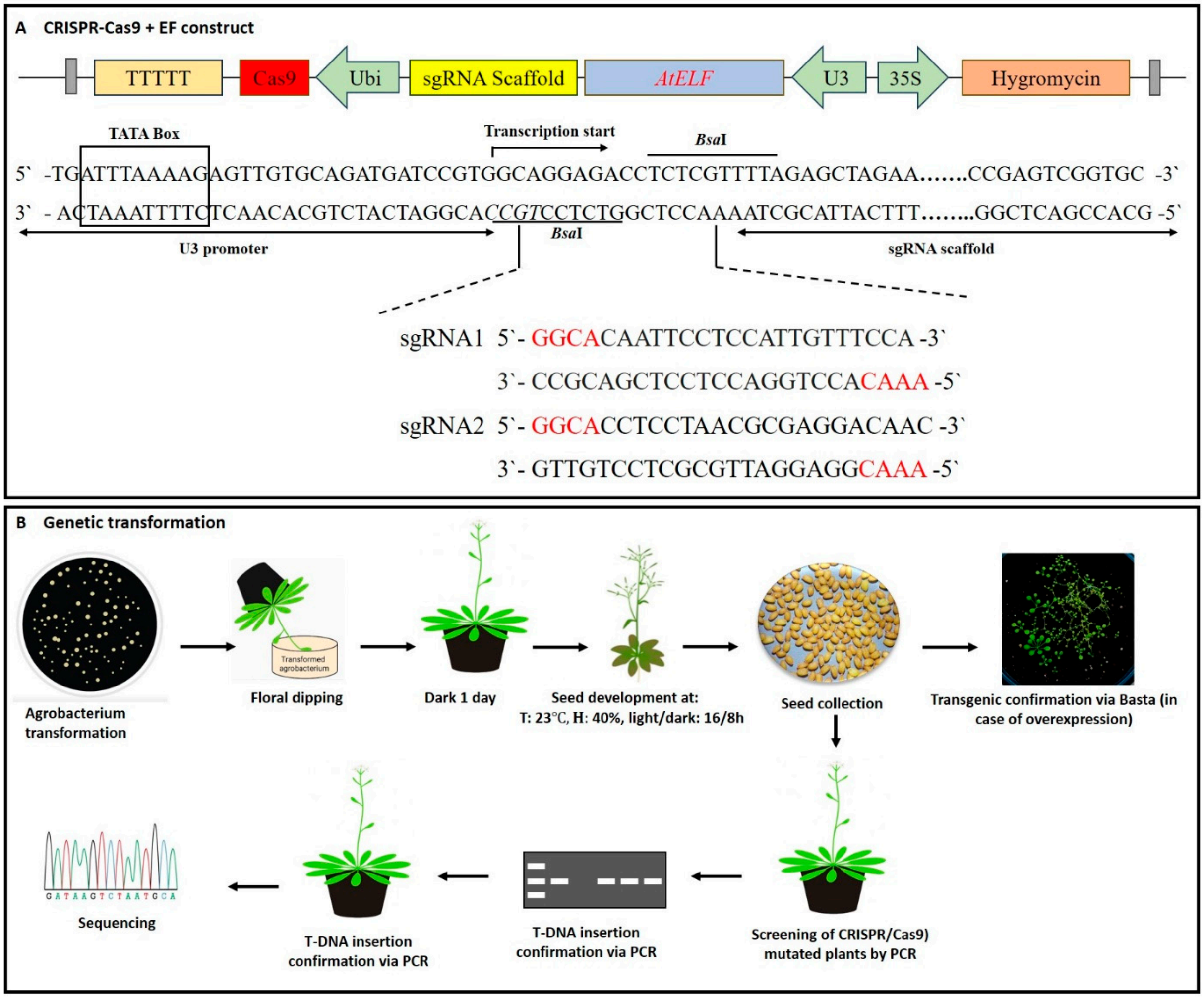

Figure S1. Schematic representation of the workflow for transgenic plant generation.

```
               Sequence (5′ to 3′)   In/Del
                     sgRNA1
Col-0  ATAACAATTCCTCCATTGTTTCCAAGGG
    1  ATAACAATTCCTCCATT-TTTCCAAGGG -1
    2  ATAACAATTCCTCCATT-TTTCCAAGGG -1
    3  ATAACAATTCCTCCATT-TTTCCAAGGG -1
    4  ATAACAATTCCTCCATTGTTTCGAAGGG C>G
    5  ATAACAATTCCTCCATTGTTTCGAAGGG C>G
```

```
               Sequence (5′ to 3′)   In/Del
                     sgRNA1
Col-0  CCCACCTCCTAACGCGAGGACAACAGTT
    1  CCCACCTCC-AACGCGAGGACAACAGTT -1
    2  CCCACCTCC-AACGCGAGGACAACAGTT -1
    3  CCCACCTCC-AACGCGAGGACAACAGTT -1
    4  CCCACCTCCTAACGCCAGGACAACAGTT G>C
    5  CCCACCTCCTAACGCCAGGACAACAGTT G>C
```

Figure S2. Target sequence genome-editing analysis for *AtELF* genome editing.
